# Bacterial Communities Associated With Spherical *Nostoc* Macrocolonies

**DOI:** 10.3389/fmicb.2019.00483

**Published:** 2019-03-21

**Authors:** Pablo Aguilar, Cristina Dorador, Irma Vila, Ruben Sommaruga

**Affiliations:** ^1^Lake and Glacier Ecology Research Group, Department of Ecology, University of Innsbruck, Innsbruck, Austria; ^2^Laboratorio de Complejidad Microbiana y Ecología Funcional, Instituto Antofagasta, Facultad de Ciencias del Mar y Recursos Biológicos, Universidad de Antofagasta, Antofagasta, Chile; ^3^Centre for Biotechnology and Bioengineering (CeBiB), Antofagasta, Chile; ^4^Departamento de Ciencias Ecológicas, Facultad de Ciencias, Universidad de Chile, Santiago, Chile

**Keywords:** cyanobacteria, bacterial diversity, Lake Chungará, Culco, 16S rRNA gene, Illumina, PICRUSt

## Abstract

Species of the genus *Nostoc* (Cyanobacteria) can form large colonies of up to several centimeters in diameter that may represent a unique habitat for bacteria in freshwaters. Bacteria inside the colony are probably segregated from the surrounding water and largely dependent on the metabolism of this primary producer. However, the existence of a specific bacterial community associated with free-living representatives of *Nostoc* from lakes and streams is unknown. Here, we studied large *Nostoc* spp. colonies (ca. 2–10 cm in diameter) from two adjacent, high altitude aquatic environments and assessed the diversity, and community composition of the bacterial community associated with the inner gelatinous matrix (GM). Further, we compared this community with that of the lake’s littoral zone where the colonies live or with the outer layer (OL) of the colony in samples collected from a stream. Alpha bacterial diversity in the inner GM of the colonies from both sites was lower than in the littoral zone or than in the OL. Significant differences in community composition were found between the inner and the OL, as well as between the inner GM, and the littoral zone. Further, these differences were supported by the putative metabolic processes of the bacterial communities. Our results indicate the existence of a specific bacterial community inside macrocolonies of *Nostoc* spp. and also imply that the inner environment exerts a strong selection. Finally, these large colonies represent not only a unique habitat, but probably also a hotspot of bacterial activity in an otherwise oligotrophic environment.

## Introduction

Interactions between bacteria and other organisms have been extensively studied in aquatic environments (e.g., Egan et al., 2013; Deveau et al., 2018; Mayali, 2018). Some well-known bacterial interactions described for the littoral zone of lakes include biofilms growing on different kind of surfaces. For example, the growth of epilithic bacteria is enhanced by the organic carbon produced and released by periphytic primary producers (Bruckner et al., 2008) and therefore, epiphytic bacterial production is substantially higher than that of planktonic bacteria (Theil-Nielsen and Sondergaard, 1999). Cyanobacteria are one of the most common primary producers found in biofilms such as in those from high elevation lakes of the temperate region (Bartrons et al., 2012), as well as in the water column of eutrophic lakes around the world (Dokulil and Teubner, 2000).

In the littoral zone of lakes and in streams, the cyanobacterium *Nostoc* sp. form colonies sometimes in high abundance (e.g., Moeller and Roskoski, 1978; Ward, 1985) that can tolerate extreme conditions such as low and high temperatures, desiccation, salt stress, and freezing (Tamaru et al., 2005; Sand-Jensen and Sand, 2012; Suradkar et al., 2017). This genus is cosmopolitan and found not only in a wide range of aquatic ecosystems, but also in terrestrial ones with some species inhabiting extreme habitats (Dodds et al., 1995; Potts, 2000; López-Cortés et al., 2001). *Nostoc* sp. is characterized by having unbranched heterocystous filaments and by the formation of gelatinous aggregates containing trichomes that, in some species, form macroscopic colonies with a range of shapes, texture and sizes (Stainer and Cohen-Bazire, 1977; Dodds et al., 1995). For example, the macroscopic colonies of *Nostoc pruniforme* Agardh can reach up to 25 cm in diameter (Potts, 2000) and form spherical colonies with an envelope of densely entangled trichomes (Dodds and Castenholz, 1987). The outer layer (OL) and inner gelatinous matrix (GM) of the colony in *Nostoc* spp. are composed of a mixture of polysaccharides (Bertocchi et al., 1990; De Philippis et al., 2000; Sand-Jensen, 2014) that protect it from a variety of environmental hazards, including high solar radiation, and that are essential for the moisture absorption and retention capacity of cells (Sand-Jensen, 2014).

Some *Nostoc* species live in symbiosis with fungi and plants (Enderlin and Meeks, 1983; Meeks, 1998; Paulsrud et al., 1998; Costa et al., 2001). For example, *Nostoc* representatives living in symbiosis with bryophytes often represent the dominant members of the N_2_-fixing bacterial community (Adams et al., 2012). Different species of *Nostoc* also establishes associations with heterotrophic bacteria such as in the case of *Nostoc flagelliforme* Calvo-Pérez & Guiry and *Nostoc commune* Bornet, É. & C. Flahault living in soils (Graham et al., 2014; Han et al., 2015; Inthasotti and Pathom-aree, 2015). For example, colonies of *N. commune* found in moist soils have a high diversity of associated Actinobacteria (Inthasotti and Pathom-aree, 2015). Whether such a specific bacterial community composition is found inside large free-living *Nostoc* colonies from lakes and streams is unknown. However, bacteria from the surrounding water are probably included during colony’s morphogenesis and if it remains intact, then the original community may shift in composition. This is plausible considering that formation of large colonies takes months (Deng et al., 2008) and that heterotrophic bacteria inside the colony would then largely depend on resources provided by this primary producer. Further, the environment inside the colony (e.g., light irradiance) is different from that in the surrounding water. For example, differences in light conditions inside colonies of *Nostoc sphaeroides* are evident when comparing the photosynthetic performance of filaments of the inner layer with those of the OL (Deng et al., 2008).

In this study, we assessed the bacterial community composition and diversity associated with macroscopic colonies of *Nostoc* spp. collected from two adjacent freshwater ecosystems located at high elevation (>4100 m above sea level) in the Andean plateau. This plateau is a region characterized by high incident UV radiation, negative water balance, and large daily temperature changes (Risacher et al., 2003; Cordero et al., 2016). First, we characterize the bacterial community composition in the inner GM of colonies found in the littoral zone of a lake and test for differences in composition with that from the surrounding littoral water. Second, we describe the bacterial community from the inner GM of colonies collected in a stream and compare it with that of the OL. We hypothesized that the different environmental conditions (e.g., light intensity and source of nutrients) between the inner part of the colony and the surrounding habitat or OL will results in a different community composition. Finally, to indirectly assess the physiology of the bacterial communities, we included a prediction analysis of their main putative metabolic processes.

## Materials and Methods

### Sampling Site

Water samples and macroscopic colonies of *Nostoc* spp. (ca. 10 cm in diameter) were collected from the littoral zone in Lake Chungará (Supplementary Figure S1A). This large lake (22.5 km^2^) is located in the Andean plateau (18°14′9.67 S, 69°10′53.81 W) at 4520 m above sea level and belongs to the Lauca National Park, a Unesco World Biosphere Reserve (Mühlhauser et al., 1995; Dorador et al., 2003). The littoral zone of this lake has an extensive area of macrophytes (e.g., *Miriophyllum elatinoides*) providing a habitat for a wide range of organisms including the endemic fish *Orestias chungarensis* Vila & Pinto and birds (e.g., *Fulica gigantea* Eydoux & Souleyet) that depends on this area for feeding and breeding (McFarlane, 1975; Vila and Pinto, 1986; Andrew, 1987). The colonies of *Nostoc* sp. are usually found at the surface of the dense submerged macrophyte belt in the littoral zone.

Samples for molecular analyses were collected from the inner part (homogenous GM) of the colony (Supplementary Figure S1B) using a sterile syringe and scalpel. A sample was obtained from one colony collected during the dry season (DS) in 2013 (sample Chungará_DS2013) and three samples were collected from three different colonies during the DS in 2016 (samples Chungará_DS2016_1, DS2016_2, and DS2016_3). A composite water sample (i.e., same volume pooled from 0.1, 0.6, and 1 m) from the littoral zone was collected with either a 2 L glass bottle (0.1 m depth) or with a 2 L Van Dorn sampler (for the other two depths) during the DS in 2013 (sample DS2013) and the wet season (WS) in 2014 (sample WS2014). Finally, one water sample was collected in 2016 during the WS (sample WS2016) and in triplicate during the DS (samples DS2016_1, DS2016_2 and DS2016_3). In addition, six colonies were collected during the DS in 2016 from the bed of a tributary stream of the Lauca River (Supplementary Figure S1C), located in “Quebrada Culco” (18°34′52.76 S, 69° 3′40.57 W, hereafter referred as to Culco stream) to compare the bacterial community of the inner GM with that of the OL. Due to the difficulty in sampling the two matrices without contamination, the sampling was done in separate colonies. Thus, three colonies were used to obtain the GM with the same methodology described above (samples Culco_DS2016_1, DS2016_2, and DS2016_3) and three other colonies were used to obtain the OL (samples Culco_DS2016_4, DS2016_5, and DS2016_6).

### Samples Processing

Water samples were kept in cold boxes and afterward (within ca. 2 h) were filtered onto 0.22 μm pore size filters (47 mm, Millipore GPWP), until clogging was observed. Filters and samples from *Nostoc* were placed in Eppendorf tubes with RNAlater (Qiagen, Germantown, MD). All samples were maintained at -20°C until further analysis.

### DNA Extraction and Illumina Sequencing

Genomic DNA was extracted using PowerBiofilm DNA Isolation kit (Mo Bio Laboratories Inc.) following the manufacturer’s protocol. The concentration and quality of DNA were measured with a Nanodrop spectrophotometer (Nanodrop 8000, Thermo Scientific). Illumina Miseq sequencing was used with two different set of primers. First, total DNA from samples in 2013 and 2014 was used as a template for the V4 region amplification of the 16S SSU rRNA with the primers 515F (GTGCCAGCMGCCGCGGTAA) and 806R (GGACTACHVGGGTWTCTAAT) (Caporaso et al., 2011), done at the Research and Testing Laboratory Genomics (Lubbock, Texas, United States). Second, samples from 2016 were used to amplify the V4-V5 region of the 16S SSU rRNA with the primers 515F-Y (GTGYCAGCMGCCGCGGTAA) and 926R (CCGYCAATTYMTTTRAGTTT) (Parada et al., 2016), done at LGC Genomics Gmbh (Berlin, Germany). The 515F-Y/926R primer improves the underestimation of SAR11 clade and the overestimation of Gammaproteobacteria produced by the 515F/806R primer (Parada et al., 2016). Raw amplicons reads were deposited in the sequence read archive (SRA) of NCBI under accession number SRP136789 and SRP136788.

### Reads Data Processing

Raw reads from 16 samples were analyzed using Mothur (v. 1.35.1) following the standard operating procedure (Schloss et al., 2011). Briefly, paired-end reads obtained with the primers 515F-Y/926R and 515F/806R were assembled using the USEARCH v.7 (Edgar and Flyvbjerg, 2014) and make.contig command, respectively. After pooling all samples and trimming the reads to the same region (V4), reads were aligned to the SILVA v.132 database using the align.seqs command. Chimeras were detected and removed using UCHIME. The SILVA v132 database was used to classify reads with a confidence threshold of 80%. The remove.lineage command was used to identify and remove mitochondrial, chloroplasts, Archaea, Eukarya, and unknown contaminants. Reads were assigned to operational taxonomic units (OTU) at the 1% level of divergence using the cluster.classic command. All OTUs with less than six reads across all samples were discarded. Samples were normalized by randomly subsampling to the same size according to the sample with the smallest number of reads. After quality control, all samples were normalized to 27157 reads. The final OTU table and sequences are available in https://doi.org/10.6084/m9.figshare.7314875.v2.

To assess alpha diversity of the bacterial communities, the Simpson and Shannon indices that combine measures of richness and abundance were calculated on equal-sized samples using the INEXT package in R (Chao et al., 2014; Hsieh et al., 2016). A Kruskal-Wallis test was made to check for significant differences (*P* < 0.05) in alpha diversity among samples grouped by sampling site. The VEGAN package (Oksanen et al., 2013) was used to do the ordinations (metaMDS) based on Bray Curtis distance using the Wisconsin square transformation of the OTU relative abundances and to test for significant differences among samples grouped by sampling site in the ordinations using ANOSIM. A maximum likelihood phylogenetic tree, using the general time reversible model (with gamma distribution and bootstrap), was constructed in RAxML v0.6.0 (Kozlov et al., 2018) with the *Nostoc* reference species (with available 16S rRNA gene) present in the database Taxonomy from the National Center for Biotechnology Information (NCBI) including one sequence from *Nostoc* sp. (Llayta) reported for the Andean plateau. Then, the short reads (OTUs) belonging to *Nostoc* spp. were mapped onto the phylogenetic tree. We also repeated the phylogenetic analysis using the database CyanoPhy (cyanobact.000webhostapp.com), however, we obtained the same results (data not shown) and thus, we present only those from the first analysis.

Calculations of the OTUs relative abundance were made excluding the OTUs classified as *Nostoc*. Samples were grouped according to the littoral water, OL from Culco and inner GM from Lake Chungará and Culco stream. A Venn diagram was created to compare genera among samples grouped by site and sample origin, based on a presence/absence matrix and visualized by the package VennDiagram in R (Chen, 2018).

The functional prediction of the bacterial communities was assessed using Phylogenetic Investigation of Communities by Reconstruction of Unobserved States (PICRUSt; Langille et al., 2013) using the galaxy server^1^. Briefly, the raw reads were re-analyzed in Mothur (v. 1.35.1) using the Greengenes v.13.5 database. All OTUs were used in downstream analyses, without including those classified as *Nostoc.* A biom file was produced with the final data and used as input for PICRUSt, where normalization by copy number, metagenome prediction and categorization by function was done. Further, the nearest sequenced taxon index (NSTI) was calculated to quantify the availability of nearby genome representatives for each microbiome sample. A low NSTI value (<0.1) indicates that the samples are highly supported by the reference microbial genome dataset (Langille et al., 2013). A principal component analysis of the predicted functions was made using STAMP v. 2.1.3 (Parks et al., 2014).

### Physico-Chemical Parameters

*In situ* measurements of water temperature and pH were done with a portable pH meter and coupled thermometer (HI9126, Hanna Instruments), whereas electrical conductivity was measured with a portable conductivity meter (Orion Star A322, Thermo Scientific). Samples were also collected in parallel for the analysis of major cations (potassium, sodium, calcium, and magnesium) by ion chromatography, and the anion, chloride was performed by the argentometric method, and sulfate by the gravimetric method (Gros, 2003). During 2013 and 2014 in L. Chungará, samples were collected in precombusted (4 h at 450°C) glass bottles for the analysis of dissolved organic carbon (DOC) and dissolved nitrogen (DN). These samples were filtered *in situ* through two pre-combusted GF/F filters (Whatman). The filtrate was acidified with HCl (pH 2) and analyzed later at the laboratory in Innsbruck, Austria with a Shimadzu TOC-Vc series equipped with a total nitrogen module. The instrument for DOC analysis was calibrated with potassium hydrogen phthalate, while calibration for the DN was done with potassium nitrate. Three to five subsamples were analyzed for each sample and for a consensus reference material (CRM) for DOC (batch 5 FS-2005: 0.57 mg; provided by RSMAS/MAC, University of Miami) that was run in parallel on each occasion. Results differed from the CRM given value by 5%, and the coefficient of variation among subsamples was <2%.

## Results and Discussion

### Bacterial Diversity and Community Patterns

**FIGURE 1:**
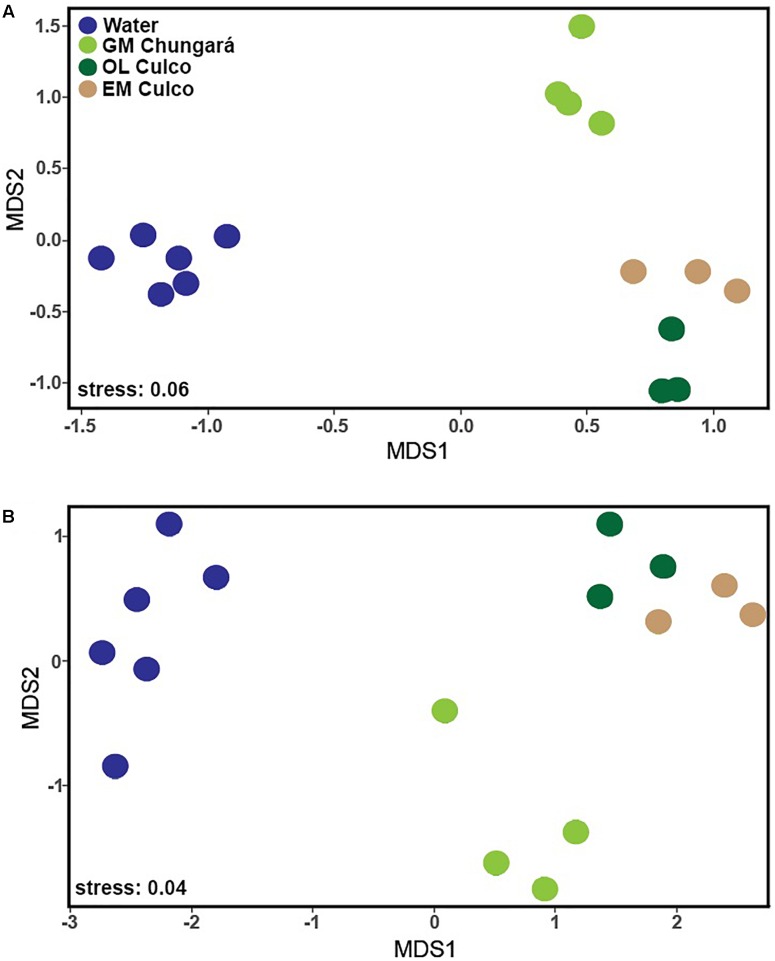
NMDS analysis of the OTUs in the samples of the littoral zone of Lake Chungará [gelatinous matrix (GM) and water] and Culco stream (GM) and outer layer (OL) with **(A)** and without **(B)** including OTUs classified as *Nostoc*. GM, gelatinous matrix; OL, outer layer.

Both alpha diversity metrics (Supplementary Figure S2) were higher for the littoral water (Shannon = 39.3 ± 13.5; Simpson = 13.9 ± 6) and for the OL (Shannon = 25 ± 4; Simpson = 12.7 ± 2.2) than for the inner GM (Lake Chungará: Shannon = 2.8 ± 1.1; Simpson = 1.5 ± 0.05 and Culco stream: Shannon = 3.6 ± 3; Simpson = 1.6 ± 0.8). Further, Shannon and Simpson indexes were significantly different between the GM and the littoral water (Kruskal-Wallis test, *P* = 0.004), as well as between the GM and the OL (*P* = 0.011). In contrast, no significant differences in diversity were found when samples of the GM were compared between Lake Chungará and the Culco stream (Kruskal-Wallis test, *P* > 0.05). An ordination analysis (based on Bray-Curtis dissimilarity) revealed that bacterial communities from the colonies collected in the Culco stream (GM and OL) were grouped close together and that differences among samples from the littoral water and the inner GM of the colonies collected from both sampling sites existed (Figure 1A). Significant differences in OTUs relative abundance (ANOSIM; *R*^2^ = 0.985, *P* < 0.001) were found among all samples. This was also true (ANOSIM; *R*^2^ = 0.875, *P* < 0.001) even after the OTUs classified as *Nostoc* were removed from the ordination analysis (Figure 1B). Overall, these results imply that the environment inside the colony selects for the bacteria probably originated from the littoral zone and included in the colony during its morphogenesis. Further, the community in the inner GM of *Nostoc* sp. from Lake Chungará appeared to be stable over time, at least at the level of taxonomic resolution analyzed (Figure 1), implying that environmental conditions inside the colony are relatively constant.

One environmental factor that is obviously different inside and outside the colony is light intensity and probably also its spectral quality. In fact, in *N. sphaeroides*, adaptation to low light levels inside the colony is clear when pigments concentration and photosynthetic performance of filaments from the inner and OL are compared (Deng et al., 2008). For example, inner filaments have a lower light saturation point, lower photosynthetic rates and efficiency, but higher chlorophyll *a* and phycobiliproteins concentrations than those from the OL (Deng et al., 2008). The large size of *Nostoc* colonies, such as those from Lake Chungará imposes constraints in the uptake of external resources and concentrations of inorganic carbon are probably also limiting inside the colony (Sand-Jensen, 2014). It remains to be tested how these differences affect the associated bacterial community.

### Bacterial Community Composition

**FIGURE 2:**
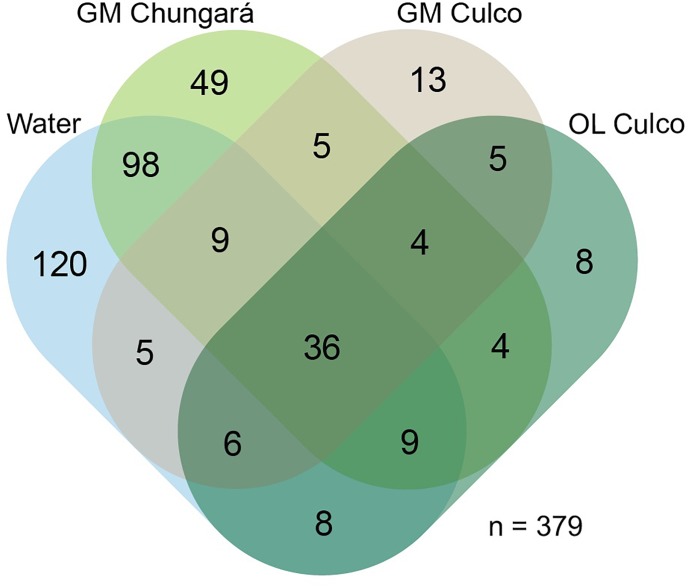
Venn diagram showing shared genera (including *Nostoc* sp.) among samples grouped by their origin: littoral lake water (Lake Chungará), inner gelatinous (Lake Chungará and Culco stream), and OL (Culco stream). GM, inner gelatinous matrix; OL, outer layer.

A total of 379 bacterial genera (24 phyla) were identified, but only 36 were shared among the littoral water, the OL and GM (Figure 2). Further, the littoral water showed the highest number of unique genera (*n* = 120) and a high number of shared genera (*n* = 98) with the GM matrix from Lake Chungará. However, few genera (*n* = 5) were shared between the GM from L. Chungará and Culco stream supporting the finding that colonies of *Nostoc* spp. from these ecosystems hold also different bacterial communities (Figure 1). This could be related with the probable existence of two different *Nostoc* species (see below discussion on identity) or with differences in bacterial community composition between the lake and the stream water included in the colony during morphogenesis, although, this needs to be tested.

**FIGURE 3:**
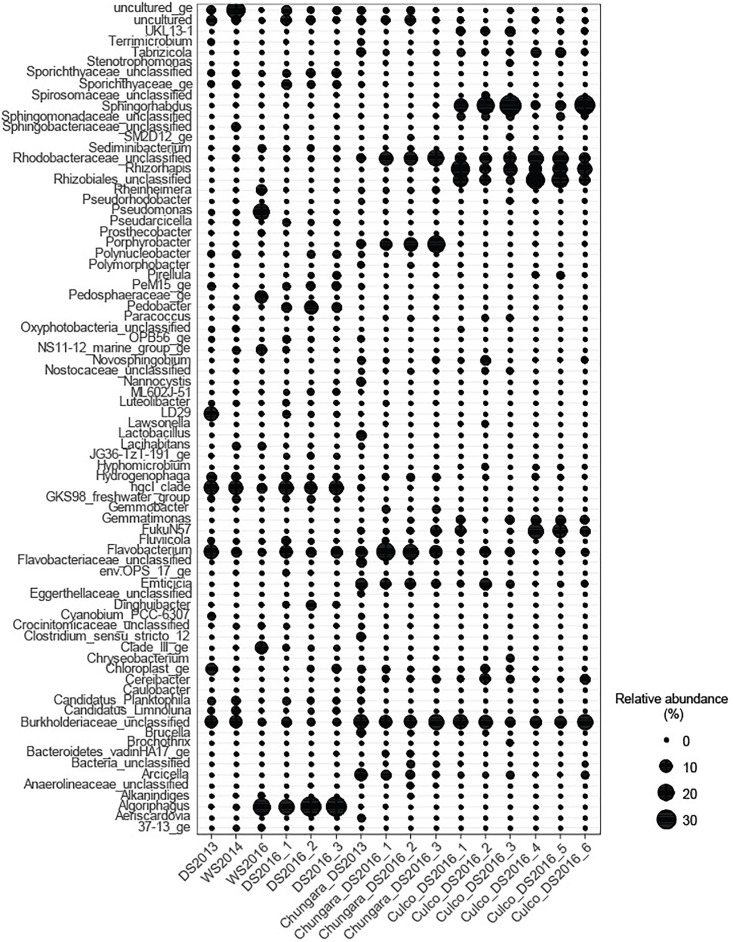
Relative abundance of the most abundant genera (>1%) in each sample. *Nostoc* was not included.

Unclassified sequences members within the Rhodobacteraceae (Alphaproteobacteria) and Burkholderiaceae (Betaproteobacteria) families were abundant (3.2–20% of relative abundance) among samples obtained from the GM and OL (Figure 3). Further, all samples from the GM of Lake Chungará showed a high relative abundance of *Porphyrobacter* sp. (Alphaproteobacteria; 2.6–22.3%), *Flavobacterium* sp. (Bacteroidetes; 7–25.1%) and *Emticicia* sp. (Bacteroidetes; 2.6–7.4%).

The GM of Culco samples showed a high relative abundance of *Sphingorhabdus* sp. (Alphaproteobacteria; 10.6–35%), *Rhizorhapis* sp. (Alphaproteobacteria; 5.6–26.7%) and unclassified Rhizobiales (Alphaproteobacteria; 2–13%). The Burkholderiaceae Family (abundant in all *Nostoc* spp. samples), *Rhizorhapis* sp. and members of Rhizobiales include an extremely diverse group of Betaproteobacteria capable of nitrogen fixation (Coenye, 2014). In addition, some of the bacterial taxa detected in the inner GM have been described for aquatic environments along the Andean plateau. For example, the order Rhodobacteriales, in our study represented by the Family Rhodobacteraceae, has been detected in association with other microorganisms in microbial mats and water samples (Dorador et al., 2013).

The main difference between the GM and OL from Culco colonies, was given by the high relative abundance of the Alphaproteobacteria FukuN57 (4.5–17.8%) in samples from the OL and the high relative abundance of the Alphaproteobacteria UKL13-1 (3.1–4.2%) in the inner GM. Interestingly, the bacterial taxa associated with *Nostoc* spp. were also common to those found in other cyanobacterial associations. For example, in *Microcystis*, a bloom-forming genus that produce mucilaginous colonies, *Porphyrobacter*, Rhodobacterales, Sphingomonadales, and Burkholderiales are also typically associated (Shi et al., 2009, 2012). Further, cyanobacterial associations with *Flavobacterium* and members of Sphingomonadaceae, Burkholderiales and Rhizobiales have been described from metagenomes of culture collections belonging to different cyanobacterial genera (Cornet et al., 2018). Similarly, *Porphyrobacter* and some members of the Family Rhodobacteraceae are known from associations with the cyanobacteria *Microcoleus* sp. (Sánchez et al., 2005), *Cylindrospermopsis* sp. (Shi et al., 2009) and *Oscillatoria brevis* Kützing (Hube et al., 2009). One of the groups found in all colonies of *Nostoc* spp. was an unclassified Burkholderiaceae. This family includes *Burkholderia*, which is not only found in association with other cyanobacteria, but also with *Mimosa* species (Angiosperm) in a nitrogen fixation symbiosis (Bontemps et al., 2010).

Despite that in the littoral water from Lake Chungará, there were no clear differences in the main physicochemical variables among seasons (Supplementary Table S1), community composition changed among samplings, though it was always clearly different from that in the inner GM (Figure 1, 3). The Hgcl clade (Actinobacteria; 3.6–13.8%) and *Flavobacterium* sp. (Bacteroidetes; 1.8–14.5%) were the predominant (high relative abundance) groups in all water samples. In addition, a high relative abundance of *Algoriphaghus* sp. (Bacteroidetes; 16.7–34%) was observed in water samples from 2016 and unclassified Burkholderiaceae (Betaproteobacteria; 10.3–10.4%) in water samples during 2013 and 2014. Comparing with the bacterial community composition from the pelagic zone of Lake Chungará, the same predominant phyla were found (Aguilar et al., 2018), though differences were observed at the genus level. For example, while *Flavobacterium* sp. (Bacteroidetes) was the dominant genus in the pelagic zone only during the DS (Aguilar et al., 2018), it was always abundant (1.8–14.3% of relative abundance) in all seasons at the littoral zone. Further, *Algoriphagus* sp. was the most relative abundant taxa in the littoral zone of Lake Chungará during the DS in 2016, but it has not been detected in the pelagic zone (Aguilar et al., 2018). Only Actinobacteria (hgcl clade) occurred in both pelagic and littoral zones at a high relative abundance.

### Predicted Metabolic Functions

**FIGURE 4:**
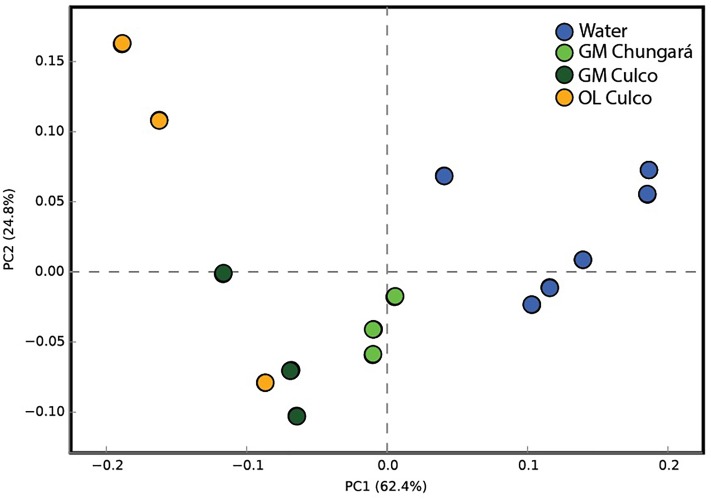
Principal component analysis of samples from the littoral zone of Lake Chungará (gelatinous matrix and water) and Culco stream (inner GM and OL) without including OTUs classified as *Nostoc* and using the predicted functions by PICRUSt. GM, inner gelatinous matrix; OL, outer layer.

The PICRUSt analysis showed a low mean NSTI value for all samples (NSTI = 0.07 ± 0.02) indicating that the predicted metabolic functions in our study were highly supported by the reference microbial genome dataset (Langille et al., 2013). The mean predicted metabolic functions of the bacterial community from the littoral zone in Lake Chungará separated well from those of the community in the inner GM (Figure 4). Thus, these results support the idea that bacteria inside and outside the colony of *Nostoc* spp. differ not only in their composition, but probably also in their physiology. However, the predictions made by PICRUSt has clear limitations (Langille et al., 2013), namely, that they are made based on the comparison between short-reads and reference genomes and thus, their interpretation should be done with caution.

### Identity of *Nostoc*

The Otu00001 and Otu00542 found in Lake Chungará were classified as *Nostoc commune*, whereas Otu00002, Otu00822 and Otu00838 found in the Culco stream were classified as *Nostoc* sp. indicating that they correspond to different species (Table 1). The Otu00001 and Otu00002 were most abundant (up to 98.5%) in Lake Chungará and Culco stream, respectively (Supplementary Table S2). The existence of two different species is also supported by the phylogenetic analysis, namely, that colonies from Lake Chungará are tentatively assigned to *Nostoc commune*, whereas those from Culco to *N. flagelliforme* (Supplementary Figure S3). Surprisingly, the sequence of *Nostoc* spp. from our study were not related to *Nostoc* sp. (Llayta), a sequence retrieved from the Andean plateau (no clear isolation source) and reported as the typical species in this area (Galetovic et al., 2017). Although the 16S rDNA gene is valid for phylogenetic studies of Cyanobacteria (Oksanen et al., 2004) and the *Nostoc* species separate well with other close cyanobacterial taxa (Svenning et al., 2005), we cannot confirm the species only based on a partial short sequence (253 bp).

**Table 1:** Taxonomical classification of OTUs (classified as *Nostoc* PCC 73102) using Genbank.

	Taxonomical classification (Genbank)					
	First hit	Coverage (%)	Identity (%)	Type strain	Coverage (%)	Identity (%)
Otu00001	*Nostoc commune* HK-02 (AP018326)	100	100	*Nostoc commune* HK-02 (AP018326)	100	100
Otu00002	*Nostoc* sp. BEA_0956 (MG543678)	100	100	*Nostoc* sp. strain 5183 (CP026692)	100	99
Otu00542	*Nostoc commune* HK-02 (AP018326)	100	99	*Nostoc commune* HK-02 (AP018326)	100	99
Otu00822	*Nostoc* sp. BEA_0956 (MG543678)	100	98	*Nostoc* sp. strain 5183 (CP026692)	100	97
Otu00838	*Nostoc* sp. BEA_0956 (MG543678)	100	98	*Nostoc* sp. strain 5183 (CP026692)	100	97


## Conclusion and Perspectives

Our results indicate that the bacterial communities associated with *Nostoc* spp. significantly differ in diversity and composition from those of the littoral zone. Overall, this study identifies these macroscopic colonies as a unique habitat for bacteria in lakes and streams and probably also as hotspots for nitrogen cycling in these aquatic ecosystems known to be N-limited (Wurtsbaugh et al., 1985). Finally, the unique bacterial community found inside large colonies of *Nostoc* spp. offers the possibility to test how autotrophic and heterotrophic microbial production are coupled. Future studies should test whether this “microcosm” is also a habitat for a unique microbial food web including predators, such as found in the balloon-like chlorophycean macroalga *Codium bursa* (Vaqué et al., 1994).

## Author Contributions

RS and PA collected the samples. PA prepared the samples for Illumina sequencing and ran the bioinformatics analysis. PA and RS wrote most of the manuscript. CD and IV contributed to the writing of the manuscript. CD, IV, and RS obtained funding for the project. All authors have read and approved this manuscript.

## Conflict of Interest Statement

The authors declare that the research was conducted in the absence of any commercial or financial relationships that could be construed as a potential conflict of interest.

## Footnotes

1 http://galaxy.morganlangille.com/

## References

[B1] Adams D. Duggan P. Jackson O. (2012). “Cyanobacterial Symbioses,” in *Ecology of Cyanobacteria II: Their Diversity in Space and Time* Vol. 593 ed. Whitton B. A. (Berlin: Springer Science+Business Media B.V), 10.1007/978-94-007-3855-3_23

[B2] Aguilar P. Dorador C. Vila I. Sommaruga R. (2018). Bacterioplankton composition in tropical high-elevation lakes of the Andean plateau. *FEMS Microbiol. Ecol.* 94:fiy004. 10.1093/femsec/fiy004 29346530 PMC6018938

[B3] Andrew T. (1987). Biological implication of lake management with reference to lago Chungará. *Arch. Biol. Med. Exp.* 20 131–134.

[B4] Bartrons M. Catalan J. Casamayor E. (2012). High bacterial diversity in epilithic biofilms of oligotrophic mountain lakes. *Microb. Ecol.* 64 860–869. 10.1007/s00248-012-0072-4 22622765

[B5] Bertocchi C. Navarini L. Cesàro A. Anastasio M. (1990). Polysaccharides from cyanobacteria. *Carbohydr. Polym.* 12 127–153. 10.1016/0144-8617(90)90015-K

[B6] Bontemps C. Elliott G. N. Simon M. F. dos Reis Junior F. B. Gross E. Lawton R. C. (2010). *Burkholderia* species are ancient symbionts of legumes. *Mol. Ecol.* 19 44–52. 10.1111/j.1365-294X.2009.04458.x 20002602

[B7] Bruckner C. Bahulikar R. Rahalkar M. Schink B. Kroth P. (2008). Bacteria associated with benthic diatoms from Lake Constance: phylogeny and influences on diatom growth and secretion of extracellular polymeric substances. *Appl. Environ. Microbiol.* 74 7740–7749. 10.1128/AEM.01399-08 18931294 PMC2607161

[B8] Caporaso J. G. Lauber C. L. Walters W. A. Berg-Lyons D. Lozupone C. Turnbaugh P. (2011). Global patterns of 16S rRNA diversity at a depth of millions of sequences per sample. *Proc. Nat. Acad. Sci. U.S.A.* 108 4516–4522. 10.1073/pnas.1000080107 20534432 PMC3063599

[B9] Chao A. Gotelli N. J. Hsieh T. C. Sander E. L. Ma K. H. Colwell R. K. (2014). Rarefaction and extrapolation with Hill numbers: a framework for sampling and estimation in species diversity studies. *Ecol. Monogr.* 84 45–67. 10.1890/13-0133.1

[B10] Chen H. (2018). *VennDiagram: Generate High-Resolution Venn, and Euler plots package., R package version 1.6.20*. Available at: https://cran.r-project.org/web/packages/VennDiagram/index.html.

[B11] Coenye T. (2014). “The Family Burkholderiaceae,” in *The Prokaryotes*, eds Rosenberg E. DeLong E. F. Lory S. Stackebrandt E. Thompson F. (Berlin: Springer).

[B12] Cordero R. Damiani A. Seckmeyer G. Jorquera J. Caballero M. Rowe P. (2016). The solar spectrum in the Atacama desert. *Sci. Rep.* 6:22457. 10.1038/srep22457 26932150 PMC4773812

[B13] Cornet L. Bertrand A. Hanikenne M. Javaux E. Wilmotte A. Baurain D. (2018). Metagenomic assembly of new (sub)polar Cyanobacteria and their associated microbiome from non-axenic cultures. *Microb. Genome* 4:e000212. 10.1099/mgen.0.000212 30136922 PMC6202449

[B14] Costa J. L. Paulsrud P. Rikkinen J. Lindblad P. (2001). Genetic diversity of *Nostoc* symbionts endophytically associated with two Bryophyte species. *Appl. Environ. Microbiol.* 67 4393–4396. 10.1128/AEM.67.9.4393-4396.2001 11526056 PMC93180

[B15] De Philippis R. Faraloni C. Margheri M. C. Sili C. Herdman M. Vincenzini M. (2000). Morphological and biochemical characterization of the exocellular investments of polysaccharide-producing *Nostoc* strains from the pasteur culture collection. *World J. Microbiol. Biotechnol.* 16 655–661. 10.1023/A:1008985722388

[B16] Deng Z. Hu Q. Lu F. Liu G. Hu Z. (2008). Colony development and physiological characterization of the edible blue-green alga, *Nostoc sphaeroides* (Nostocaceae, Cyanophyta). *Prog. Nat. Sci.* 18 1475–1483. 10.1016/j.pnsc.2008.03.031

[B17] Deveau A. Bonito G. Uehling J. Paoletti M. Becker M. Bindschedler S. (2018). Bacterial–fungal interactions: ecology, mechanisms and challenges. *FEMS Microbiol. Rev.* 42 335–352. 10.1093/femsre/fuy008 29471481

[B18] Dodds W. Castenholz R. (1987). Effects of grazing and light on the growth of *Nostoc pruniforme* (Cyanobacteria). *Br. Phycol. J.* 23 219–227. 10.1080/00071618800650251

[B19] Dodds W. Gudder D. Mollenhauer D. (1995). The ecology of *Nostoc*. *J. Phycol.* 31 2–18. 10.1111/j.0022-3646.1995.00002.x

[B20] Dokulil M. Teubner K. (2000). Cyanobacterial dominance in lakes. *Hydrobiologia* 438 1–12. 10.1023/A:1004155810302

[B21] Dorador C. Pardo R. Vila I. (2003). Temporal variations of physical, chemical and biological parameters of a high altitude lake: the case of Chungará lake. *Rev. Chil. Hist. Nat.* 76 15–22.

[B22] Dorador C. Vila I. Witzel K. P. Imhoff J. F. (2013). Bacterial and archaeal diversity in high altitude wetlands of the Chilean Altiplano. *Fund. Appl. Limnol.* 182 135–159. 10.1127/1863-9135/2013/0393

[B23] Edgar R. C. Flyvbjerg H. (2014). Error filtering, pair assembly and error correction for next-generation sequencing reads. *Bioinformatics* 31 3476–3482. 10.1093/bioinformatics/btv401 26139637

[B24] Egan S. Harder T. Burke C. Steinberg P. Kjelleberg S. Thomas T. (2013). The seaweed holobiont: understanding seaweed–bacteria interactions. *FEMS Microbiol. Rev.* 37 462–476. 10.1111/1574-6976.12011 23157386

[B25] Enderlin C. Meeks J. (1983). Pure culture and reconstitution of the *Anthoceros-Nostoc* symbiotic association. *Planta* 158 157–165. 10.1007/BF00397709 24264545

[B26] Galetovic A. Araya J. Gómez-Silva B. (2017). Biochemical composition and toxicity of edible colonies of the cyanobacterium *Nostoc* sp. *Llayta. Rev. Chil. Nutr.* 44 360–370. 10.4067/s0717-75182017000400360

[B27] Graham L. Knack J. Piotrowski M. Wilcox L. Cook M. Wellman C. (2014). Lacustrine *Nostoc* (Nostocales) and associated microbiome generate a new type of modern clotted microbialite. *J. Phycol.* 50 280–291. 10.1111/jpy.12152 26988185

[B28] Gros N. (2003). Ion chromatographic analyses of sea waters, brines and related Samples. *Water* 5 659–676. 10.3390/w5020659

[B29] Han P. Shen S. Jia S. Wang H. Zhong C. Tan Z. (2015). Comparison of bacterial community structures of terrestrial cyanobacterium *Nostoc flagelliforme* in three different regions of China using PCR-DGGE analysis. *World J. Microbiol. Biotechnol.* 31 1061–1069. 10.1007/s11274-015-1856-8 25940326

[B30] Hsieh T. C. Ma K. H. Chao A. (2016). iNEXT: an r package for interpolation and extrapolation of species diversity (Hill numbers). *Methods Ecol. Evol.* 7 1451–1456. 10.1111/2041-210X.12613

[B31] Hube A. Heyduck-Söller B. Fischer U. (2009). Phylogenetic classification of heterotrophic bacteria associated with filamentous marine cyanobacteria in culture. *Syst. Appl. Microbiol.* 32 256–265. 10.1016/j.syapm.2009.03.001 19423262

[B32] Inthasotti T. Pathom-aree W. (2015). Diversity of Actinobacteria associated with *Nostoc* *commune* Voucher ex Bornet & Flahault macrocolonies. *Ann. Microbiol.* 65 2229–2240. 10.1007/s13213-015-1063-8

[B33] Kozlov A. Darriba D. Flouri T. Morel B. Stamatakis A. (2018). RAxML-NG: a fast, scalable, and user-friendly tool for maximum likelihood phylogenetic inference. *bioRxiv* [Preprint]. 10.1101/447110 PMC6821337 31070718

[B34] Langille M. Zaneveld J. Caporaso J. McDonals D. Knights D. Reyes J. (2013). Predictive functional profiling of microbial communities using 16S rRNA marker gene sequences. *Nat. Biotechnol.* 31 814–821. 10.1038/nbt.2676 23975157 PMC3819121

[B35] López-Cortés A. García-Pichel F. Nübel U. Vázquez-Juárez R. (2001). Cyanobacterial diversity in extreme environments in Baja California, Mexico: a polyphasic study. *Int. Microbiol.* 4 227–236. 10.1007/s10123-001-0042-z 12051567

[B36] Mayali X. (2018). Editorial: metabolic interactions between bacteria and phytoplankton. *Front. Microbiol.* 9:727. 10.3389/fmicb.2018.00727 29692774 PMC5902574

[B37] McFarlane R. (1975). Notes on the giant Coot (*Fulica gigantea*). *Condor* 77 324–327. 10.2307/1366228

[B38] Meeks J. (1998). Symbiosis between nitrogen-fixing cyanobacteria and plants. *BioScience* 48 266–276. 10.2307/1313353

[B39] Moeller R. Roskoski J. (1978). Nitrogen-fixation in the littoral benthos of an oligotrophic lake. *Hydrobiologia* 60 13–16. 10.1007/BF00018682

[B40] Mühlhauser H. Hrepic N. Mladinic P. Montecino V. Cabrera S. (1995). Water quality and limnological features of a high altitude Andean lake, Chungará, in northern Chile. *Rev. Chil. Hist. Nat.* 68 341–349.

[B41] Oksanen I. Lohtander K. Sivonen K. Rikkinen J. (2004). Repeat-type distribution in trnL intron does not correspond with species phylogeny: comparison of the genetic markers 16S rRNA and trnL intron in heterocystous cyanobacteria. *Int. J. Syst. Evol. Microbiol.* 54 765–772. 10.1099/ijs.0.02928-0 15143022

[B42] Oksanen J. Blanchet F. G. Kindt R. Legendre P. McGlinn D. Friendly M. (2013). *Vegan: Community Ecology Package., R Package Version 2.0-7*. Available at: http://CRAN.R-project.org/package=vegan

[B43] Parada A. Needham D. M. Fuhrman J. A. (2016). Every base matters: assessing small subunit rRNA primers for marine microbiomes with mock communities, time series and global field samples. *Environ. Microbiol.* 18 1403–1414. 10.1111/1462-2920.13023 26271760

[B44] Parks D. Tyson G. Hugenholtz P. Beiko R. (2014). STAMP: statistical analysis of taxonomic and functional profiles. *Bioinformatics* 30 3123–3124. 10.1093/bioinformatics/btu494 25061070 PMC4609014

[B45] Paulsrud P. Rikkinen J. Lindblad P. (1998). Cyanobiont specificity in some *Nostoc*-containing lichens and in a *Peltigera aphthosa* photosymbiodeme. *New Phytol.* 139 517–524. 10.1046/j.1469-8137.1998.00220.x

[B46] Potts M. (2000). “Nostoc,” in *Ecology of Cyanobacteria: Their Diversity in Time and Space*, eds Whitton B. A. Potts M. (Dordrecht: Kluwer Academic Publishers), 151.

[B47] Risacher F. Alonso H. Salazar C. (2003). The origin of brines and salts in chilean salars: a hydrochemical review. *Earth Sci. Rev.* 63 249–293. 10.1016/S0012-8252(03)00037-0

[B48] Sánchez O. Diestra E. Esteve I. Mas J. (2005). Molecular characterization of an oil-degrading cyanobacterial consortium. *Microb. Ecol.* 50 580–588. 10.1007/s00248-005-5061-4 16341637

[B49] Sand-Jensen K. (2014). Ecophysiology of gelatinous *Nostoc* colonies: unprecedented slow growth and survival in resource-poor and harsh environments. *Ann. Bot.* 114 17–33. 10.1093/aob/mcu085 24966352 PMC4071103

[B50] Sand-Jensen K. Sand T. (2012). Tolerance of the widespread cyanobacterium *Nostoc commune* to extreme temperature variations (-269 to 105 °C), pH and salt stress. *Oecologia* 169 331–339. 10.1007/s00442-011-2200-0 22120705

[B51] Schloss P. Gevers D. Westcott S. (2011). Reducing the effects of PCR amplification and sequencing artifacts on 16S rRNA-based studies. *PLoS One* 6:e27310. 10.1371/journal.pone.0027310 22194782 PMC3237409

[B52] Shi L. Cai Y. Kong F. Yu Y. (2012). Specific association between bacteria and buoyant *Microcystis* colonies compared with other bulk bacterial communities in the eutrophic lake Taihu, China. *Environ. Microbiol. Rep.* 4 669–678. 10.1111/1758-2229.12001 23760939

[B53] Shi L. Cai Y. Yang H. Xing P. Li P. Kong L. (2009). Phylogenetic diversity and specificity of bacteria associated with *Microcystis aeruginosa* and other cyanobacteria. *J. Environ. Sci.* 21 1581–1590. 10.1016/S1001-0742(08)62459-6 20108694

[B54] Stainer R. Y. Cohen-Bazire G. (1977). Phototrophic prokaryotes: the Cyanobacteria. *Ann. Rev. Micrbiol.* 31 225–274. 10.1146/annurev.mi.31.100177.001301 410354

[B55] Suradkar A. Villanueva C. Gaysina L. Casamatta D. Saraf A. Dighe G. (2017). *Nostoc thermotolerans* sp. nov., a soil-dwelling species of *Nostoc* (Cyanobacteria). *Int. J. Syst. Evol. Microbiol.* 67 1296–1305. 10.1099/ijsem.0.001800 28109209

[B56] Svenning M. Eriksson T. Rasmussen U. (2005). Phylogeny of symbiotic cyanobacteria within the genus *Nostoc* based on 16S rDNA sequence analyses. *Arch. Microbiol.* 183 19–26. 10.1007/s00203-004-0740-y 15549268

[B57] Tamaru Y. Takani Y. Yoshida T. Sakamoto T. (2005). Crucial role of extracellular polysaccharides in desiccation and freezing tolerance in the terrestrial cyanobacterium *Nostoc commune*. *Appl. Environ. Microbiol.* 71 7327–7333. 10.1128/AEM.71.11.7327-7333.2005 16269775 PMC1287664

[B58] Theil-Nielsen J. Sondergaard M. (1999). Production of epiphytic bacteria and bacterioplankton in three shallow lakes. *Oikos* 86 283–292. 10.2307/3546446

[B59] Vaqué D. Agustí S. Duarte C. M. Enríquez S. Geertz-Hansen O. (1994). Microbial heterotrophs within *Codium bursa*: a naturally isolated microbial food web. *Mar. Ecol. Prog. Ser.* 109 275–282. 10.3354/meps109275

[B60] Vila I. Pinto M. (1986). A new species of killifish (Pisces, Cyprinodontidae) from the Chilean Altiplano. *Rev. Hydrobiol. Trop.* 19 233–239.

[B61] Ward A. (1985). Factors affecting distribution of *Nostoc* in cascade mountain streams of western Oregon, U. S. A. Verh. *lnternat. Verein. Limnol.* 22 2799–2804. 10.1080/03680770.1983.11897777

[B62] Wurtsbaugh W. A. Vincentr W. F. Alfaro R. Vincent C. L. Richerson P. J. (1985). Nutrient limitation of algal growth and nitrogen fixation in a tropical alpine lake, Lake Titicaca (Peru/Bolivia). *Freshwat. Biol.* 15 185–195. 10.1111/j.1365-2427.1985.tb00191.x

