# Supplementary material for: Bacterial Communities Associated With Spherical Nostoc Macrocolonies
Source: Front Microbiol. 2019 Mar 21;10:483. doi: 10.3389/fmicb.2019.00483 (PMC6437075; doi:10.3389/fmicb.2019.00483)
Supplement: Supplementary file 1 [file Data_Sheet_1.docx]

Supplementary Material

**Bacterial communities associated with spherical *Nostoc* macrocolonies**

***Pablo Aguilar^1*^, Cristina Dorador^2,3^, Irma Vila^4^, and Ruben Sommaruga^1^***

^1^Lake and Glacier Ecology Research Group, Department of Ecology, University of Innsbruck, Austria.

^2^ Laboratorio de Complejidad Microbiana y Ecología Funcional, Instituto Antofagasta, & Facultad de Ciencias del Mar y Recursos Biológicos, Universidad de Antofagasta, Antofagasta, Chile.

^3^ Centre for Biotechnology and Bioengineering (CeBiB), Antofagasta, Chile.

^4^ Departamento de Ciencias Ecológicas, Facultad de Ciencias, Universidad de Chile, Santiago, Chile.

*** Correspondence**:

Pablo Aguilar, Lake and Glacier Ecology Research Group, Department of Ecology, University of Innsbruck, Technikerstr. 25, 6020 Innsbruck, Austria. pablo.aguilar-espinosa@student.uibk.ac.at.

**Table S1.** Main physico-chemical parameters of the littoral zone from Lake Chungará and of Culco stream. Values are the mean for measurements made in triplicates ± 1 S.D. when available. DS: Dry season. WS: Wet season

|  | **Lake Chungará (Littoral zone)** | | | |  | **Culco stream** |
| --- | --- | --- | --- | --- | --- | --- |
|  | DS2013 | WS2014 | WS2016 | DS2016 |  | DS2016 |
| Temperature [°C] | 8.9 (NA) | NA | NA | 7.6 (±0.5) |  | 18 (±0) |
| pH | 9.63 (NA) | NA | 8.7(±0.2) | 9.1 (±0.2) |  | 8.2 (±0) |
| Dissolved oxygen [mg L^-1^] | 8.4 (NA) | NA | 5.4 (±0.3) | 5.9 (±0.2) |  | 5.6 (±0.15) |
| Conductivity [μS cm^-1^] | 1456 (NA) | NA | 1220 (±179.6) | 1540 (±62.9) |  | 229 (±1.4) |
| NO_3_^-^-N [mg L^-1^] | 0 (NA) | 0.005 (NA) | NA | 0.0007 (±0.08) |  | 0.0001 (±0) |
| SO_4_^2-^ [mg L^-1^] | 111.6 (NA) | 104.4 (±9.7) | 82.8 (±11.4) | 122.3 (±11.54) |  | 23.4 (±0.6) |
| Cl^-^ [mg L^-1^] | 61.609 (NA) | 62.05 (NA) | 1.4 (±0) | 70.9 (±0) |  | 17.7 (±0) |
| Na^+^ [mg L^-1^] | 140.154 (NA) | 142.1 (±0.8) | 30.9 (±4.8) | 183.8 (±8.2) |  | 15 (±0.8) |
| K^+^ [mg L^-1^] | 28.951 (NA) | 32.3 (±2.7) | 29.9 (±1.2) | 27.4 (±0.9) |  | 3.1 (±0.1) |
| Mg^2+^ [mg L^-1^] | 99.265 (NA) | 101.7 (±1.7) | 48 (±9.1) | 87.4 (±8.8) |  | 7 (±0.7) |
| Ca^2+^ [mg L^-1^] | 55.746 (NA) | 47.2 (±6.2) | 41.5(±3.05) | 51.5 (±4.4) |  | 19.8 (±0.2) |
| DOC [mg L^-1^] | 11.2 (NA) | 11.2 (NA) | NA | NA |  | NA |
| DN [mg L^-1^] | 0.94 (NA) | 0.95 (NA) | NA | NA |  | NA |
| NA: not available |  |  |  |  |  |  |

**Table S2.** Relative abundance (%) of the five OTUs classified as *Nostoc* found in water samples from the littoral zone of Lake Chungará and in colonies of *Nostoc* spp. from Lake Chungará and Culco stream.

|  |  |  |  | | |  |  |  |  |  |  |  |  |  |  |  |  |  |  |  |  |  |
| --- | --- | --- | --- | --- | --- | --- | --- | --- | --- | --- | --- | --- | --- | --- | --- | --- | --- | --- | --- | --- | --- | --- |
|  |  | **Water samples from L. Chungará** | | | | | | | |  | **Colonies from Lake Chungará** | | | |  | **Colonies from Culco stream** | | | | | |  |
|  |  | **DS2013** | | **WS2014** | | **WS2016** | **DS2016_1** | **DS2016_2** | **DS2016_3** |  | **DS2013** | **DS2106_1** | **DS2106_2** | **DS2106_3** |  | **DS2016_1** | **DS2016_2** | **DS2016_3** | **DS2016_4** | **DS2016_5** | **DS2016_6** |  |
|  | **Otu00001** | 0.04 | | | 0.01 | 0 | 0 | 0 | 0 |  | 98.5 | 81.7 | 83.3 | 80.3 |  | 0.01 | 0.01 | 0.03 | 0 | 0 | 0 |  |
|  | **Otu00002** | 0.003 | | | 0 | 0 | 0 | 0 | 0 |  | 0 | 0.01 | 0.02 | 0.01 |  | 61.5 | 93.4 | 91.08 | 0.11 | 0.003 | 0.73 |  |
|  | **Otu00542** | 0 | | | 0 | 0 | 0 | 0 | 0 |  | 0 | 0.03 | 0.01 | 0.01 |  | 0 | 0 | 0 | 0 | 0 | 0 |  |
|  | **Otu00822** | 0 | | | 0 | 0 | 0 | 0 | 0 |  | 0 | 0 | 0 | 0 |  | 0 | 0 | 0.02 | 0 | 0 | 0 |  |
|  | **Otu00838** | 0 | | | 0 | 0 | 0 | 0 | 0 |  | 0 | 0 | 0 | 0 |  | 0 | 0 | 0.02 | 0 | 0 | 0 |  |
|  |  |  | | |  |  |  |  |  |  |  |  |  |  |  |  |  |  |  |  |  |  |

**Fig S1**  A, Littoral zone of Lake Chungará. B, Colonies of *Nostoc* sp. from Lake Chungará. C, Colonies of *Nostoc* sp. from Culco stream.

**
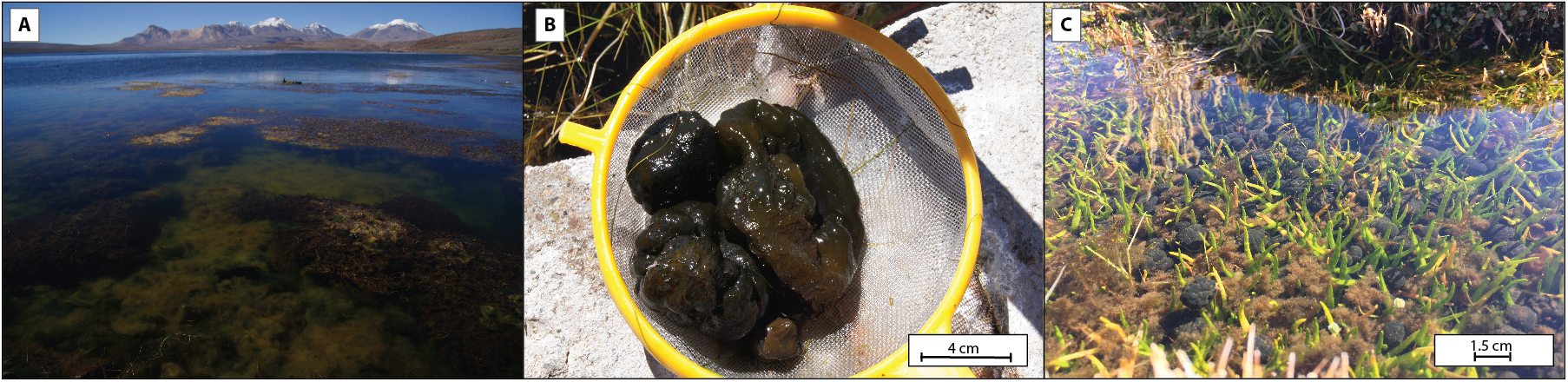
**

**Fig S2** Shannon and Simpson Diversity among samples grouped by sampling site. GM: inner gelatinous matrix. OL: outer layer.


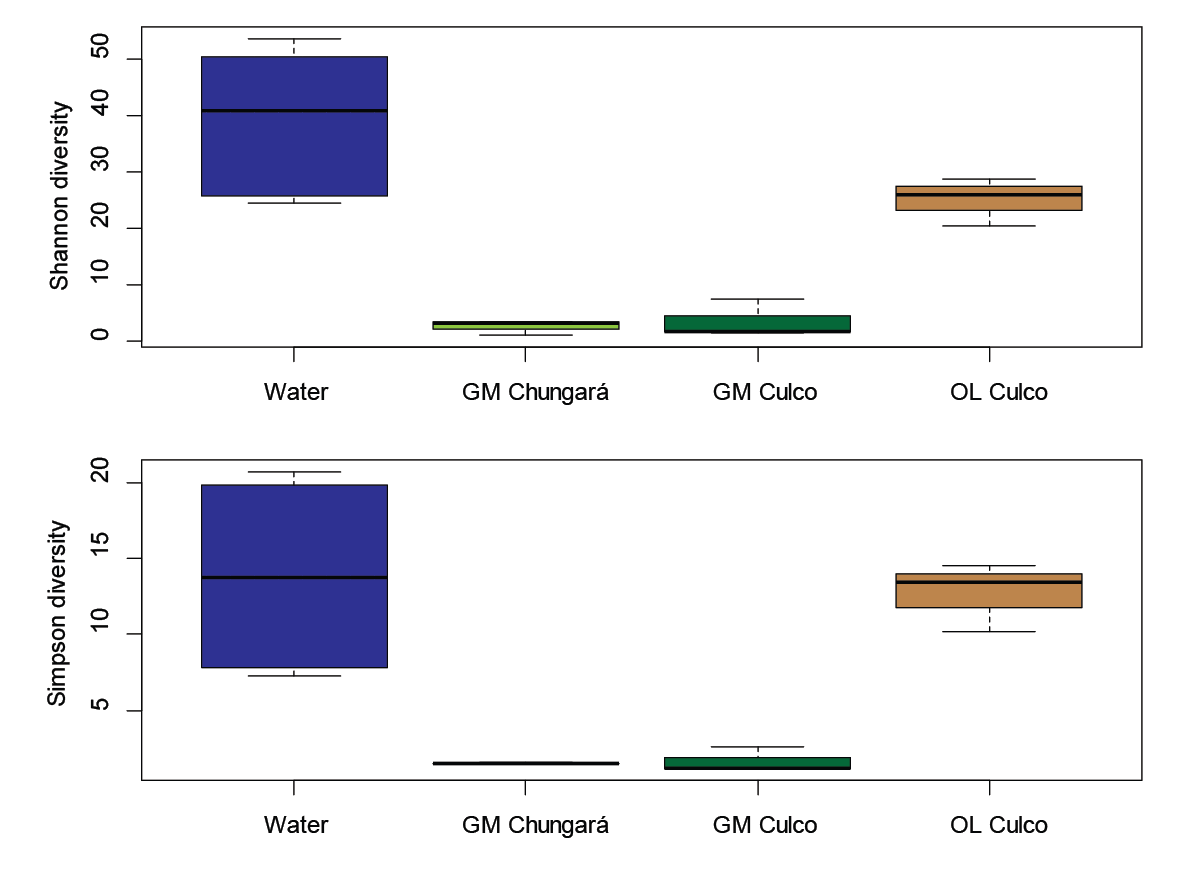


**Fig S3** Phylogenetic tree based on partial 16S rRNA gene sequences of *Nostoc* reference species and the OTUs from this study classified as *Nostoc* spp. (red) and a sequence from *Nostoc* sp. from the Andean plateau (blue). The tree was produced by Maximum likelihood using the general time reversible model, gamma distribution and bootstrap. The root corresponds to the 16S rRNA from *Synechococcus* *elongatus*. The scale bar represents the changes per nucleotide position. GenBank accession number for each sequence is shown within brackets.
